# Age-Restriction of a Validated Risk Scoring Tool Better Predicts HIV Acquisition in South African Women: CAPRISA 004

**DOI:** 10.1007/s10461-022-03664-y

**Published:** 2022-04-13

**Authors:** Delivette Castor, Emma K. Burgess, Nonhlanhla Yende-Zuma, Craig J. Heck, Quarraisha Abdool Karim

**Affiliations:** 1 Division of Infectious Diseases, Department of Medicine, Columbia University Irving Medical Center, New York, NY USA; 2 Independent Consultant, Toronto, Canada; 3 Centre for the AIDS Programme of Research in South Africa (CAPRISA), University of KwaZulu-Natal, Private Bag X7, Congella, Durban, 4013 South Africa; 4 Department of Epidemiology, Mailman School of Public Health, Columbia University, New York, NY USA

**Keywords:** HIV, Prevention, Risk Score, PrEP, South Africa

## Abstract

*We examined the predictive ability of the VOICE risk screening tool among adolescent girls and young women at heightened HIV risk in urban and peri-urban Kwa-Zulu-Natal, South Africa.* Using participant data from CAPRISA 004’s control arm (N = 444), we applied the initial VOICE risk screening score (IRS), a modified risk score (MRS) based on predictive and non-predictive variables in our data, and age-restricted (AIRS and AMRS, respectively). We estimated incidence rates, 95% confidence bounds, sensitivity, specificity, negative and positive predictive values and area under the curve (AUC). The sample’s HIV incidence rate was 9.1/100 Person-Years [95% CI 6.9–11.7], resulting from 60 seroconversions (60/660.7 Person-Years). The IRS’ ≥ 8 cutpoint produced moderate discrimination [AUC = 0.66 (0.54–0.74), sensitivity = 63%, specificity = 57%]. Restricting to age < 25 years improved the score’s predictive ability (AIRS: AUC = 0.69, AMRS: AUC = 0.70), owing mainly to male partner having other partners and HSV-2. *The risk tool predicted HIV acquisition at a higher cutpoint in this sample than in the initial VOICE analysis. After age-stratification, fewer variables were needed for maintaining score’s predictiveness. In this high incidence setting, risk screening may still improve the efficiency or effectiveness of prevention counseling services. However, PrEP should be offered to all prevention-seeking individuals, regardless of risk ascertainment.*

**Supplementary Information:**

The online version contains supplementary material available at 10.1007/s10461-022-03664-y.

## Introduction

**Fig. 1:**
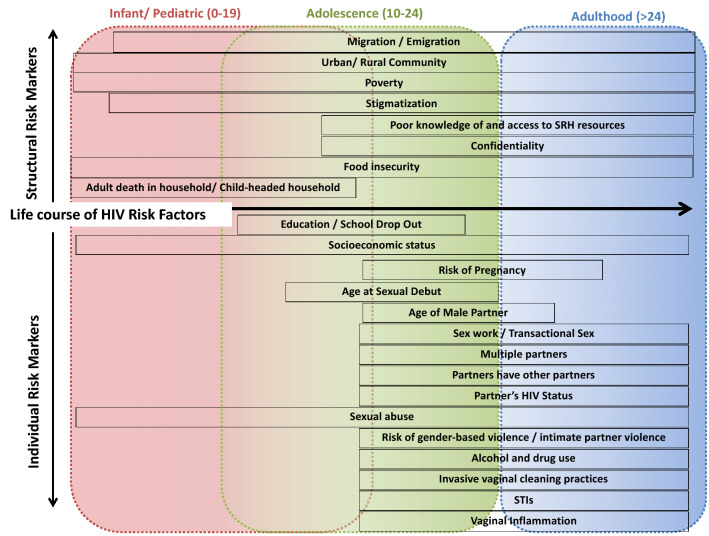
Individual- and structural-level risk factors throughout the life course for South African women. Adapted from Dellar, Waxman, and Abdool Karim (http://www.samj.org.za/index.php/samj/article/view/10099)

HIV prevention for women, particularly adolescent girls and young women (AGYW, aged 15–24 years), remains critical for ending the HIV epidemic [1]. AGYW contributes to approximately 25% of all seroconversions in sub-Saharan Africa (SSA). In South Africa, AGYW has a prevalence that is eight-fold of their male counterparts [2, 3]. Several individual- and structural-level biological, behavioral, and social factors (Fig. 1) may amplify AGYW’s HIV risk in South Africa [3–5].

The armamentarium of biomedical HIV prevention technologies for AGYW is expanding. The World Health Organization (WHO) currently recommends tenofovir-based daily oral pre-exposure prophylaxis (PrEP) and conditionally recommends the monthly dapivirine ring (DVR) for women as an additional prevention choice for people at substantial HIV risk [6]. The HIV prevention trials network (HPTN) 084 preliminary efficacy results showed long-acting Cabotegravir injections reduced HIV transmission by 90% compared to oral PrEP use [HR 0.11 (0.04–0.32)] [7]. Global HIV prevention targets within the joint WHO/UNAIDS fast track goals for ending the HIV epidemic fell far short of 3.1 million people on PrEP by 2020, and 2025 prevention targets are even more ambitious [8]. To accelerate prevention efforts, provide choice, allocate prevention according to population needs and the health system requirements for sustained use of biomedical prevention, the field needs to identify effective and efficient ways to deliver HIV prevention to AGYW in a stigma-free way [9–12].

HIV risk assessment tools have been increasingly explored as an approach to identify people who need HIV prevention efficiently. Still, the tool’s effectiveness may vary, and some argue that these tools may perpetuate stigma [13–17]. Balkus et al*.* developed one of the few validated tools for women, the VOICE risk score, in the context of HIV biomedical prevention trials conducted in Eastern and Southern Africa. The voice tool includes seven risk factors, and at a cutpoint of 3, moderately predicted substantial risk of HIV acquisition, defined by the WHO as HIV incidence ≥ 3% per year [16]. External validation of the VOICE risk score for oral PrEP showed findings from null in a lower HIV incidence setting to comparably moderate in other settings with similar HIV incidence [16–21]. In this higher HIV incidence setting of KwaZulu-Natal, South Africa, we assessed the external performance of the VOICE risk score using CAPRISA 004 (CAP004) data, a sample of primarily AGYW, and modified the tool based on our data. We examined how the risk scoring tool could be simplified while improving its predictive ability.

## Methods

### Study Design and Population

The methods and results of CAPRISA 004 were described elsewhere [22]. Briefly, this was a phase IIb, double-blind, randomized, placebo-controlled efficacy trial of 1% tenofovir gel that enrolled women (N = 889) in KwaZulu-Natal, South Africa, between May 2007–March 2010. Eligible participants were aged 18–40 years, HIV negative, sexually active, non-pregnant, and non-barrier contraceptive users. Eligible participants who demonstrated adequate understanding of the trial, assessed through a comprehension checklist, were enrolled after providing written informed consent. Randomization was 1:1 ratio to the intervention (1% tenofovir gel) or placebo. Study staff provided comprehensive HIV prevention counseling at all visits and collected demographic and behavioral data. Given the intervention’s protective effect, we conduct this analysis using the placebo arm only (n = 444) [22].

### Ethical Considerations

The study (NCT00441298) was approved by the University of KwaZulu-Natal’s Biomedical Research Ethics Committee (E111/06), Family Health International’s Protection of Human Subjects Committee (#9946), the South African Medicines Control Council (#20060835), and Columbia University (#AAAT3256).

### External Validation and Modification of Initial Risk Score (IRS)

We used the variables and methodology reported by Balkus et al*.* to create and externally validate the VOICE initial risk score (IRS). The IRS included the following variables or their proxy measures: age (< 25 years/ ≥ 25 years), financial or material support (partner/other), married or cohabiting (yes/no), primary male partner has other partners (yes/no/don’t know), alcohol use in the last three months (yes/no), laboratory diagnosis of curable STI symptoms (yes/no), and herpes simplex virus type 2 (HSV-2) seropositivity (yes/no) [16]. Three IRS variables were defined differently in the CAPRISA 004 study: alcohol use was defined as pre-coital alcohol use in the last 30 days; partner exclusivity was defined as any male partner having other partners; STI diagnosis was based on self-report of vaginal discharge. Participants’ IRS totaled 11 (Supplemental Table 2). We examined associations between HIV incidence and variables included in the IRS and additional factors within the CAPRISA data (e.g., contraception, number of casual partners). A modified risk score (MRS) was developed to include additional variables associated with HIV in our sample, and we examined if the MRS improved the predictive ability for AGYW. Since nearly 80% of incident HIV infections occurred among women < 25 years, age was stratified for IRS and MRS and were called AIRS and AMRS, respectively. Only the young women stratum aged < 25 years was analyzed. In the unadjusted analysis of women aged > 25 years old, no variable was associated with HIV incidence rate, as determined by inclusion of the null within the 95% confidence intervals. Further, the confidence intervals were wide, and some parameter estimates did not achieve convergence, indicating that the number of events was too few to analyze further. Balkus et al. described that we created an additional modified risk score [public health risk score (PHRS)] without HSV-2 since laboratory testing is not routinely available in many low-resource settings. AMRS and PHRS produced different scores based on the approach described by Balkus et al*.,* individual predictors included in the final model were assigned a score by dividing the coefficient for the predictor in the final model by the lowest coefficient among all predictors in the model and rounding to the nearest integer.

### Statistical Analysis

For each model, IRS, MRS, AIRS AMRS, and PHRS, we used Cox proportional hazards to analyze the univariate and multivariate relationships between the variables included in each model and HIV acquisition among participants with complete data. Only variables with confidence intervals excluding the null were included in the multivariate model producing the final score in modified risk scores. The Akaike information criterion and likelihood ratio tests were used to compare each model of the IRS model. We evaluated risk score performance by calculating HIV incidence per 100 PY (person-years) and generating receiver operating characteristics (ROC) of the area under the curve (AUC) to explore the prediction of the total risk score. We calculated 95% confidence intervals of ROC using bootstrap methods [23]. We also calculated the time-dependent sensitivity and specificity of risk scores categorized at ≥ 3 and ≥ 5 and determined the optimal cutpoint for our population-based on HIV incidence differences [16]. The negative (NPV) and positive predictive values (PPV) were calculated as an extension of the time-dependent sensitivity and specificity, using the equations: PPV = true positive/risk score positive; NPV = true negative/risk score negative. We calculated time-dependent ROC curves using SurvialROC and SensSpec packages within R (version 3.4.0) and performed all other analyses using Stata (version 13).

## Results

There were 60 HIV seroconversions in the CAP004 control arm cohort over 660.7 PY [incidence rate (Ir) = 9.1/100 PY (6.9–11.7)]. Table 1 shows the distribution and unadjusted association variables in the IRS (detailed definitions in Supplementary Table 1) and additional variables examined for the MRS associated with HIV incidence in this sample. There were 58 seroconversions included in the IRS with 641.7 PY (Ir = 9.0/100PY), and two observations were dropped due to missingness. Most participants (N = 444) were younger than 25 years (68.02%), received income from sources other than their partner (87.61%), lived away from their partner (87.61%), were uncertain (61.89%), or certain that partners had other partners (21.09%), experienced abnormal vaginal discharge (30.63%), had HSV-2 (49.32%) and drank alcohol pre-coitally in the last 30 days (34.23%) (Table 1). In addition, 83.56% used injectable contraceptives, 12.84% reported casual sex partnerships, and 85.14% were monogamous (Table 1). In the unadjusted analyses of the variables in the VOICE IRS model, only partners had other partners was associated with HIV in the entire sample. Among women < 25 years, HSV-2 was also associated (Table 1). In this dataset, we also found use of injectable contraception [HR = 2.86 (1.04–7.88)] and casual partnerships [HR = 1.91 (1.02–3.60)] were associated with HIV risk in the unadjusted analysis (Table 1). After adjustment, age < 25 years [aHR = 2.47 (1.24–4.89)], uncertainty [aHR = 4.02 (1.22–13.30)] and certainty that male partners had other partners [aHR = 3.77 (1.07–13.30)], and HSV-2 seropositivity [aHR = 2.10 (1.19–3.68)] were associated with increased HIV risk in IRS (Table 2). The association between HIV and uncertainty [aHR = 9.43 (1.25–71.17)] and certainty [aHR = 10.65 (1.36–83.26)] that partners had other partners and HSV-2 [aHR = 2.48 (1.35–4.58)], was even stronger among women < 25 in the AIRS (Table 2). Table 2 also showed that after adjustments for potential confounding factors in our dataset (MRS), contraception was no longer associated, but casual partnerships [aHR = 2.19 (1.09–4.39)] remained associated with HIV acquisition. A similar trend was observed in women < 25 years (Table 2, AMRS). In the PHRS model, after adjustment, certainty [aHR = 8.84 (1.16)] that partner had other partners and having casual partnerships [aHR = 2.10 (1.06–4.16)] was associated with HIV seroconversion (Table 2).

**Table 1:** Univariable distribution of sample characteristics and estimates from Cox proportional hazards regression

Factors	Baseline characteristics (n = 444)	Full sample (n = 444)	Women < 25 years (n = 302)	Women ≥ 25 years (n = 142)
	Proportion (%)	HR (95% CI)	HR (95% CI)	HR (95% CI)
IRS factors				
Age				
< 25	68.02	1.74 (0.94–3.22)		
≥ 25 (Ref)	31.98	1.00		
Income source				
Partner (Ref)	12.39	1.00	1.00	1.00
Other	87.61	2.00 (0.73–5.52)	1.77 (0.55–5.69)	2.34 (0.30–17.97)
Married or living with				
Partner (Ref)	12.39	1.00	1.00	1.00
Parents or other	87.61	1.06 (0.48–2.33)	0.46 (0.17–1.29)	1.38 (0.38–5.01)
Partners have other partners				
No (Ref)	15.01	1.00	1.00	1.00
Don't Know	61.89	3.43 (1.06–11.10)	6.84 (0.93–50.20)	1.60 (0.35–7.29)
Yes	23.09	3.85 (1.12–13.20)	9.92 (1.31–75.10)	0.41 (0.04–4.50)
Combined (Don’t know/Yes)^a^	84.99	3.54 (1.11–11.31)	7.63 (1.05–55.36)	1.26 (0.30–5.69)
Abnormal vaginal discharge				
No (Ref)	69.37	1.00	1.00	1.00
Yes	30.63	0.97 (0.56–1.69)	1.12 (0.60–2.06)	0.63 (0.17–2.29)
HSV-2 positive				
No (Ref)	50.68	1.00	1.00	1.00
Yes	49.32	1.54 (0.92–2.60)	2.50 (1.40–4.48)	0.60 (0.18–1.96)
Alcohol use before sex last 30 days				
No (Ref)	65.77	1.00	1.00	1.00
Yes	34.23	1.26 (0.75–2.11)	1.72 (0.96–3.06)	0.57 (0.17–1.85)
Additional factors explored in CAP004				
Contraception				
Other (Ref)	16.44	1.00	1.00	–
Injectables	83.56	2.86 (1.04–7.88)	1.48 (0.53–4.11)	–
Casual partners in last year				
None (Ref)	87.16	1.00	1.00	1.00
≥ 1	12.84	1.91 (1.02–3.60)	2.39 (1.22–4.70)	0.64 (0.08–4.95)
Total number of partners in last year				
1 (Ref)	85.14	1.00	1.00	1.00
> 1	14.86	1.74 (0.94–3.22)	2.24 (1.16–4.31)	0.50 (0.06–3.84)

Analyses include variables comprising the VOICE initial risk score and other variables explored within the CAPRISA004 dataset

Dashes (–) represent non-convergence due to small sample size

< 3% missingness for each variable

Additional factors that were assessed, but had null findings, are presented in Supplemental Table 3

*HR* cox proportional hazard ratio, *95% CI* 95% confidence interval

^a^Categorization only used in CART analysis

**Table 2:** Multivariable analyses of factors associated with HIV acquisition, modeling the Initial risk score (IRS), modified risk score (MRS), < 25 age modified risk score (< 25 AMRS), and public health risk score (PHRS)

	IRS model	< 25 AIRS	≥ 25 AIRS	MRS model	< 25 AMRS model	PHRS model
	aHR (95% CI)	aHR (95% CI)	aHR (95% CI)	aHR (95% CI)	aHR (95% CI)	aHR (95% CI)
Age						
< 25	2.47 (1.24–4.89)			2.22 (1.16–4.25)		
≥ 25 (Ref)	1.00			1.00		
Income source						
Partner (Ref)	1.00	1.00	1.00			
Other	2.26 (0.81–6.30)	2.49 (0.75–8.27)	2.51 (0.31–20.16)			
Married or living with						
Partner (Ref)	1.00	1.00	1.00			
Parents or other	0.67 (0.28–1.59)	0.43 (0.15–1.26)	1.25 (0.33–4.75)			
Partners have other partners						
No (Ref)	1.00	1.00	1.00	1.00	1.00	1.00
Don't Know	4.02 (1.22–13.25)	9.43 (1.25–71.17)	1.40 (0.30–6.65)	3.65 (1.12–11.80)	7.56 (1.03–55.70)	6.45 (0.88–47.32)
Yes	3.77 (1.07–13.33)	10.65 (1.36–83.26)	0.32 (0.03–3.79)	3.53 (1.02–12.20)	7.86 (1.02–60.30)	8.84 (1.16–67.25)
Abnormal discharge						
No (Ref)	1.00	1.00	1.00			
Yes	1.09 (0.62–1.91)	1.28 (0.69–2.40)	0.95 (0.24–3.79)			
HSV-2 seropositive						
No (Ref)	1.00	1.00	1.00	1.00	1.00	
Yes	2.10 (1.19–3.68)	2.48 (1.35–4.58)	0.80 (0.24–2.71)	2.07 (1.19–3.62)	2.54 (1.39–4.63)	
Alcohol use before sex last 30 days						
No (Ref)	1.00	1.00	1.00			
Yes	1.31 (0.77–2.23)	1.61 (0.89–2.94)	0.60 (0.18–2.09)			
Casual partners in last year						
None (Ref)					1.00	1.00
≥ 1					2.19 (1.09–4.39)	2.10 (1.06–4.16)
AIC	678.76	484.29	131.84	675.13	480.92	499.20

Despite its association in univariate Cox regression, injectable contraceptive use was not included in the MRS model due to convergence issues

Number of partners in the last year was not included in < 25 AMRS model due to collinearity with casual partners in the last

*aHR* adjusted cox proportional hazards ratio, *95% CI* 95% confidence intervals, *IRS* initial risk score, *MRS* modified risk score, *AMRS* age modified risk score, *PHRS* public health risk score, *AIC* akaike information criterion

### IRS Validation and AIRS

**Fig. 2:**
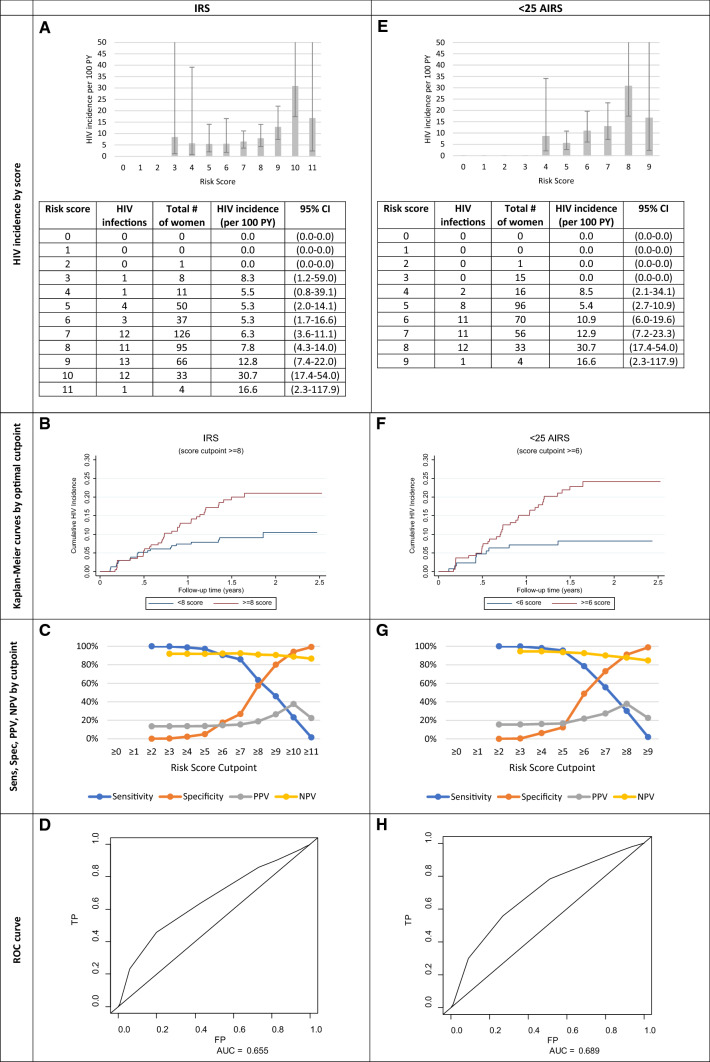
Measures of HIV incidence and diagnostic accuracy for the initial risk score (IRS, n = 431) and age-stratified initial risk score [AIRS, aged < 25 years (n = 291)]. *Sens* sensitivity, *Spec* specificity, *PPV* positive predictive value, *NPV* negative predictive value, *ROC* receiver operating characteristic, *TP* true positive, *FP* false positive, *AUC* area under curve. The IRS and AIRS were created using individuals with complete information for all the factors

The mean IRS was 7 (range: 2–11), and HIV incidence increased as risk scores increased (Fig. 2A). The > 3 and ≥ 5 cutpoints previously reported by Balkus et al*.* were indiscriminate in this sample, with incidence rates of 8.3/100 PY and 5.2/100 PY, respectively. Only one woman had a score of < 3, and 4.4% had scores of < 5, which explains the high sensitivity of 100% and 97%, but lower specificity of 0.3% and 5% observed, respectively. A cutpoint ≥ 8 (> 8: Ir = 12.8/100 PY vs. < 8: Ir = 6.0/100 PY) was found to be optimal (Fig. 2B), with but its discrimination was moderate [sensitivity: 63%, specificity: 57% (Fig. 2C)], identifying 64% of infections among 46% of the population. The IRS’ predictive power was moderate [AUC: 0.66 (0.54–0.74), Fig. 1D]. Exactly 97.07% (N = 431) participants had complete data on the 7 IRS items used throughout the analyses. They did not differ from the entire sample. There were no factors predicting HIV seroconversion in women ≥ 25 years, so all age-stratified scores report women < 25 years. Figure 2E shows young women (aged < 25 years) had 47 seroconversions [Ir = 10.7/100 PY (7.9–14.1)]. The optimal cutpoint of > 6 highlighted a difference in HIV incidence (> 6: Ir = 15.12/100 PY vs. < 6: Ir = 5.07/100 PY), had a 78% sensitivity and 49% specificity (Fig. 2F, G) and identified 78% of infections among 56% of this population. The AIRS slightly improved predictive power [AUC: 0.69 (0.60–0.78)] than the IRS (Fig. 2H), but there was still no threshold that met the WHO substantial risk guideline [24].

### Alternative Risk Scoring Tools: MRS, AMRS, and PHRS

**Fig. 3:**
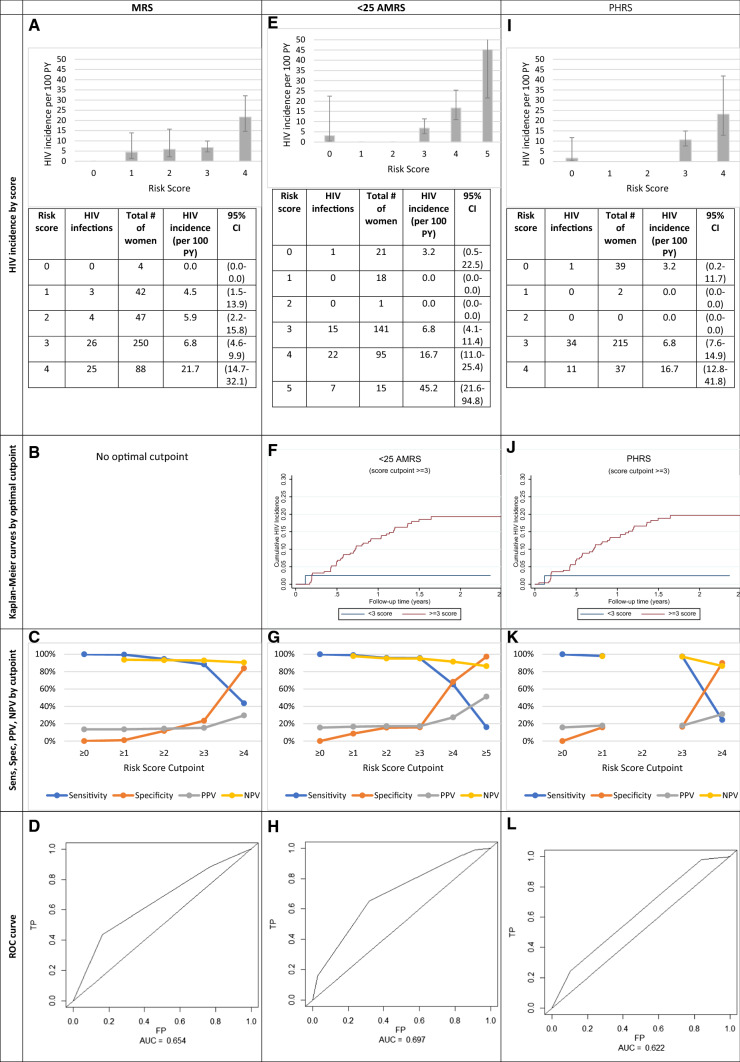
Measures of HIV incidence and diagnostic accuracy for the modified risk score (MRS, n = 431), < 25 Age-stratified MRS (< 25 ARMS, n = 291) and Public Health Risk Score (PHRS, n = 293). *Sens* sensitivity, *Spec* specificity, *PPV* positive predictive value, *NPV* negative predictive value, *ROC* receiver operating characteristic, *TP* true positive, *FP* false positive, *AUC* area under curve. The MRS and PHRS were created using individuals with complete information for all the factors. There is a small sample size difference between the < 25 AMRS and PHRS because we were able to reincorporate 2 individuals who did not have HSV-2 data

Relative to the IRS, where the AUC was observed to be 0.66, the AUC for the MRS, AMRS and PHRS were 0.65, 0.70, and 0.62, respectively (Figs. 2, 3). Showing the best predictive ability, those with an AMRS of < 3 (13.8%) had an incidence rate [1.61/100 PY (0.23–11.40)], over seven-times smaller than those with > 3 scores [12.01/100 PY (8.93–16.13), Fig. 3E] At the optimal cutpoint of 4 in the AMRS, HIV Ir = 16.7. The sensitivity and specificity of this cutpoint were 95.5% and 15.8%, respectively (Fig. 3G, H). The PHRS (2–3 vs. 6/7 factors), which excluded the lab measurement not typically conducted in clinical settings, HSV-2 could predict risk similarly to the IRS. Those with < 3 PHRS had a lower incidence [1.57/100 PY (0.22–11.16)] compared with those who had > 3 scores [12.58/100 PY (9.17–16.44)] (Fig. 3I).

## Discussion

The HIV incidence observed in this cohort, 9.1/100 PY overall and 10.7 in women < 25 years, represents a unique hyper-epidemic scenario to examine the VOICE risk assessment tool (IRS). The IRS for the entire sample and when restricted to AGYW < 25 years (AIRS) showed moderate discrimination, 61, 69% sensitivity, and 57% specificity for both models, respectively. While the AUC was comparable to the findings reported by Balkus et al*.*, the risk threshold > 3 or > 5 was indiscriminate for HIV acquisition in this study [16, 18]. IRS and AIRS had a threshold of ≥ 8 and > 6, respectively. Women with IRS < 8 had approximately two-fold lower HIV incidence (5.95% vs. 12.8%). Another external validation among young women in a lower HIV incidence setting did not find the VOICE risk assessment to have predictive ability [19]. In a comparative analysis of the VOICE tool and another risk assessment tool developed by Ayton et al*.*, the VOICE score had a lower performance in observed and simulated data and had different risk thresholds [17, 25]. Balkus et al. reported comparable performance of the risk assessment tool in the ASPIRE trial sample where HIV Ir was 3.7%, and only a subset of variables in risk tool was available [21]. Peebles et al*.* examined the predictive ability of the score among women in the ECHO trial where HIV Ir was 3.9% overall. Peebles further examined whether age-restriction improved a modified score’s predictive ability in women 18–24 and 25–35 years old, where HIV Ir was 5.4 and 3.4% and found no meaningful difference in AUC within and across age bands [20]. The optimal thresholds in the younger and older sub-groups were > 5 and > 6, corresponding to HIV Ir of 8.5 and 8.6%, respectively.

In our dataset, two of the eight VOICE risk factors were consistently associated with HIV acquisition after adjustment in all risk models: HSV-2 seropositivity and knowing or being unsure that male partners had other partners. The predictive ability of a risk score containing just these two variables in the entire sample (AUC = 0.60) of women < 25 years (AUC = 0.68) did not differ meaningfully from the IRS containing all variables and was corroborated in an exploratory classification and regression trees (Supplemental Figs. 2, 3). Casual partnership, the only variable associated with HIV in our dataset that was not part of the VOICE risk score **w**hen added to the MRS model containing partner has other partners, and HSV-2 also maintained predictive ability (AUC = 0.66, Supplemental Fig. 3). Others have reported the effect of HSV-2 as a risk classifier. Still, in the absence of routine testing and clinical decision-making based on the diagnosis, the public health utility of this assay remains debatable [20].

Our analysis has some strengths and limitations. First, the CAP004 trial collected baseline STI status through self-report and syndromic management instead of laboratory testing, and alcohol use was pre-coital and in the last 30 days, compared with three months in the VOICE score. The prevalence of STIs in CAP004 compared to the VOICE validation samples was 30.63% and 20.00%, respectively, highlighting the potential magnitude of misclassification. While these definitional differences could have influenced our observed findings, a sensitivity analysis excluding these proxy variables did not change the AUC (AUC = 0.66 vs. 0.67 with excluded proxies: data not shown). Other external validation studies have reported similar challenges with differences in measurement of risk score factors [17, 19, 21]. Uniformity in measurement will improve the tool’s reliability over time and settings. Second, our sample size was smaller than used in previous validations, and the results are susceptible to potential biases and reduced power. However, the number of observed incident HIV cases was high and comparable to larger cohorts, which improved the power of this cohort. The stratified analysis of women < 25 years improved the predictive ability but reduced the score’s generalizability to the broader population. We pursued age-stratified risk scores because vulnerabilities in younger women may differ from those of older women. The multivariate analysis among older women did not produce statistically reliable estimates due to too few events. The collapsed estimates may not be representative of risk among older women. Empiric data from KwaZulu Natal showed that HIV incidence is gradually increasing in women > 25 years as effective interventions are scaled among the youth [26]. More than 40% of incident infections occurred among women < 25 years despite this incidence shift. As the HIV epidemic evolves, incidence changes, and understanding HIV incidence data across age, sex, gender, or other markers of HIV vulnerability, will help prioritize demographic groups for interventions to lower transmission. The 2007–2010 incidence rates for sub-districts Vulindlela and eThekwini, where the CAPRISA 004 trial was implemented, were 11.20/100PY-15.60/100PY [27, 28]. Recent district-level population-level estimates showed lower rates overall but still elevated among women aged 20–24 years and ranks among some of the highest rates globally (4.26/100PY) [29]. In the current epidemic context, risk screening is being applied to improve the efficiency of HIV prevention trial recruitment. Concomitantly, risk screening is being applied to improve efficiency and cost-effectiveness in PrEP delivery in health services. Using the VOICE risk cutpoint > 3, all but one woman would have been eligible for trial recruitment or PrEP services. The inability of the risk tool to discriminate at the threshold of substantial HIV risk in this sample may limit its utility in higher-risk settings like KwaZulu-Natal. Our findings may have resulted from the successful enrollment of high-risk women into CAP004 and would have more predictive ability in a broader sample of women. Possibly, universal PrEP use in young women may be more impactful in high incidence settings like urban and rural KwaZulu Natal. Two variables resulted in similar predictive ability as the entire VOICE risk score. Applying a more straightforward risk tool is less time and resource-intensive in environments like ours and may improve program and recruitment efficiencies.

We were only able to look at women 18 years of age and older. Given the high incidence rate in women aged 18–19-years-old [Ir = 6.63/100 PY (3.45–12.74)] in this sample, some AGYW < 18 years may already be at substantial HIV risk. They also have unique risk characteristics and should be prioritized for PrEP services and participation in prevention trials. Using relevant variables from the VOICE score, the ‘Ayton’ risk score was the first to estimate HIV risk in AGYW < 18 years across high, low, and no risk classifications and demonstrated relatively higher sensitivity and specificity [17]. Age stratification also revealed that women ≥ 25 years of age had an incidence rate exceeding 6%, with five of the nine total infections occurring in the 26–27 age band. However, the sample size was too small to explore this further. AGYW remains a vital group for HIV prevention; however, these findings highlight the need to understand who remains at HIV risk in other age bands of women and why.

## Conclusion

The age-restricted modified risk score (AMRS) demonstrated comparable predictive ability as the VOICE risk score, but not at the same optimal cutpoints. HIV risk scores commonly include age as a variable even though it is a non-modifiable risk factor [15, 16, 30–33]. As biomedical prevention options for AGYW increase, understanding the modifiable factors that drive risk within age bands is critically essential for comprehensive and tailored HIV prevention (Fig. 1). In this sample, two variables produced comparable predictive ability overall and among women < age 25, HSV-2, and whether male partners had other partners. The partner dynamics within relationships continue to elude interventionists [34–37]. Age-specific counseling in unstable partnerships should be an essential part of comprehensive prevention [38–40]. HSV-2 status has been associated with HIV in this and other studies but remains un-intervenable [41, 42]. Most clinical settings do not currently offer and likely will not offer HSV testing as part of their routine HIV counseling, and treatment for risk reduction remains unproven [43, 44]. The PHRS examined the model’s predictiveness without HSV-2 and found it moderately predictive (AUC = 0.62), though considerably less than the AIRS and AMRS, which included HSV-2 among women age < 25. We emphasize that the age restriction requires additional validation; however, we encourage other studies focusing on PrEP delivery to consider disaggregating risk factors by narrower age bands.

## Supplementary Information

Below is the link to the electronic supplementary material.

Supplementary file1 (DOCX 169 KB)

## Data Availability

The data used in this analysis are publicly available (upon request) on CAPRISA’s website.
