# Supplementary material for: Age-Restriction of a Validated Risk Scoring Tool Better Predicts HIV Acquisition in South African Women: CAPRISA 004
Source: AIDS Behav. Author manuscript; Available in PMC 2022 Oct 1. (PMC9474358; doi:10.1007/s10461-022-03664-y)
Supplement: 1801140_Sup_Info [file NIHMS1801140-supplement-1801140_Sup_Info.docx]

**Supplementary Material: Age-restriction of a validated risk scoring tool better predicts HIV acquisition in South African women: CAPRISA 004**

**Supplemental Table 1.** Variable definitions

| **Variable** | **Category** | **Definition** |
| --- | --- | --- |
| Age | <25 years | Reported being 24 years old or younger |
|  | ≥25 years | Reported being 25 years old or older |
| Sources of income | Partner | Listed husband/stable partner listed as income source |
|  | Other | Listed self-generated income, salary/wage, social grants/family support, no income, or other as income source(s) |
| Married/cohabiting | Yes | Responded “married” when asked about their relationship status or said that they were living with their regular partner. |
|  | No | Responded “stable, casual, or no partner” when asked about their relationship status and said they did not live with their regular partner or did not have a regular partner. |
| Partners have other partners | Yes | In last 30 days, believed any sexual partner(s) had other sexual partners |
|  | Don’t know | In last 30 days, did not know if any sexual partner(s) had other sexual partners |
|  | No | In last 30 days, did not believe any sexual partner(s) had other sexual partners |
| Alcohol use | Yes | In the last 30 days, respondent or partner consumed alcohol before sex. |
|  | No | In the last 30 days, respondent or partner did not consume alcohol before sex. |
| Curable STI symptoms | Yes | Reported vaginal discharge at baseline |
|  | No | Did not report vaginal discharge at baseline |
| HSV-2 seropositivity | Yes | Technologists confirmed serostatus using Kalon Enzyme Immunoassay (Kalon Biologicals, Ashgate, UK) |
|  | No | Technologists confirmed serostatus using Kalon Enzyme Immunoassay (Kalon Biologicals, Ashgate, UK) |
| Contraception | Injectables | Reported using Depo Provera or Nuristerate |
|  | Other | Reported using other methods (oral contraceptives, diaphragm, IUD, emergency contraceptives, condoms, rhythm method, thigh sex, withdrawal, spermicide, tubal ligation, hysterectomy, vasectomy, or traditional method) |
| Casual partners in last year | None | No reported casual partners in last year |
|  | ≥1 | At least 1 casual partner in the last year |
| Total number of partners in last year | 1 | 1 stable or casual partner in the last year |
|  | >1 | More than 1 stable or casual partner in the last year |
| Rural | Yes | Rural study site |
|  | No | Urban study site |
| Completed high school | Yes | Completed high school, tertiary education, or some tertiary education |
|  | No | Completed primary school, some primary school or high school, or no schooling at all |
| Parity | No children | No reported live births |
|  | ≥1 | At least 1 reported live birth |
| Ever exchange sex for money | Yes | Ever received money/gifts in exchange for sex |
|  | No | Never received money/gifts in exchange for sex |
| Sexual debut age | ≤17 | Sexually debuted at age 17 or younger |
|  | >17 | Sexually debuted at age 18 or older |
| Partner age difference | ≤5 years | In the last 30 days, oldest sexual partner was ≤5 years older |
|  | >5 years | In the last 30 days, oldest sexual partner was >5 years older |
| Condom frequency | Always | Always used condoms during sex |
|  | Inconsistently | Occasionally, most times, or never used condoms during sex |
| Time lived in Durban area | <1 year | Lived in the Durban area for less than 1 year |
|  | ≥1 year | Lived in the Durban area for at least 1 year |
| Knowledge of a partner testing HIV positive in last 30 days | Know partners status as positive or Don’t know | Responded “Yes” or “ Don’t know” when asked if any recent (i.e., within the last 30 days) sexual partner(s) ever tested positive for HIV |
|  | Know partners status as negative | Responded “No” when asked if any recent (i.e., within the last 30 days) sexual partner(s) ever tested positive for HIV |

**Supplemental Table 2.** Composition of risk scores

| **Predictor** | **Category** | **Initial Risk Score**  **(IRS)** | **Age-stratified IRS**  **(AIRS)** | | **Modified Risk Score (MRS)** | **Age Modified Risk Score (AMRS)** | **Public Health Risk Score**  **(PHRS)** |
| --- | --- | --- | --- | --- | --- | --- | --- |
|  |  |  | **<25 years** | **>25 years** |  | **<25 years** |  |
| **Sample size**^†^ |  | **n=431** | **n=291** | **n=140** | **n=431** | **n=291** | **n=293** |
| Age | <25 | 2 |  |  | 1 |  |  |
|  | ≥25 | 0 |  |  | 0 |  |  |
| Income source | Partner | 0 | 0 | 0 |  |  |  |
|  | Other | 1 | 1 | 1 |  |  |  |
| Married or living with | Partner | 0 | 0 | 0 |  |  |  |
|  | Parents or other | 2 | 2 | 2 |  |  |  |
| Partners have other partners | No | 0 | 0 | 0 | 0 | 0 | 0 |
|  | Don’t know | 2 | 2 | 2 | 2 | 3 | 3 |
|  | Yes | 2 | 2 | 2 | 2 | 3 | 3 |
| Abnormal discharge | No | 0 | 0 | 0 |  |  |  |
|  | Yes | 1 | 1 | 1 |  |  |  |
| HSV-2 seropositive | No | 0 | 0 | 0 | 0 | 0 |  |
|  | Yes | 2 | 2 | 2 | 1 | 1 |  |
| Alcohol use before sex in last 30 days | No | 0 | 0 | 0 |  |  |  |
|  | Yes | 1 | 1 | 1 |  |  |  |
| Casual partners in last year | None |  |  |  |  | 0 | 0 |
|  | ≥1 |  |  |  |  | 1 | 1 |
| **Total score** |  | **11** | **9** | **9** | **4** | **5** | **4** |

There is a small sample size difference between the AMRS and PHRS because we were able to reincorporate 2 individuals who did not have HSV-2 data

^†^ Sample sizes represents individuals with complete cases

**Supplemental Table 3.** Additional factors explored in CAP004 dataset with null (p≥0.05) univariable statistical associations

| Factors | Baseline  Characteristics  (n=444) | Full sample  (n=444) | Women <25 years  (n=302) | Women ≥25 years  (n=142) |
| --- | --- | --- | --- | --- |
|  | Proportion (%) | HR (95% CI) | HR (95% CI) | HR (95% CI) |
| Additional factors explored in CAP004 | |  |  |  |
| Rural |  |  |  |  |
| No (Ref) | 31.76 | 1.00 | 1.00 | 1.00 |
| Yes | 68.24 | 1.02 (0.59-1.77) | 0.87 (0.46-1.62) | 1.34 (0.41-4.36) |
| Completed high school |  |  |  |  |
| No (Ref) | 58.33 | 1.00 | 1.00 | 1.00 |
| Yes | 41.67 | 1.13 (0.68-1.88) | 1.20 (0.68-2.13) | 0.80 (0.25-2.61) |
| Parity |  |  |  |  |
| No children (Ref) | 24.10 | 1.00 | 1.00 | 1.00 |
| ≥1 | 75.90 | 0.86 (0.48-1.52) | 1.02 (0.54-1.90) | 0.65 (0.14-2.93) |
| Ever exchange money for sex |  |  |  |  |
| No (Ref) | 97.75 | 1.00 | 1.00 | - |
| Yes | 2.25 | 1.68 (0.41-6.91) | 3.10 (0.75-12.78) | - |
| Sexual debut >17 |  |  |  |  |
| No (Ref) | 51.02 | 1.00 | 1.00 | 1.00 |
| Yes | 48.98 | 0.67 (0.40-1.12) | 0.64 (0.35-1.17) | 1.01 (0.33-3.10) |
| Partner Age Difference |  |  |  |  |
| <5 years (Ref) | 77.70 | 1.00 | 1.00 | 1.00 |
| >5 years | 22.30 | 1.29 (0.72-2.33) | 1.33 (0.68-2.58) | 1.12 (0.31-4.10) |
| Condom frequency |  |  |  |  |
| Always (Ref) | 29.50 | 1.00 | 1.00 | 1.00 |
| Inconsistently | 70.50 | 1.30 (0.72-2.32) | 1.37 (0.71-2.65) | 1.18 (0.33-4.31) |
| Time lived in Durban area |  |  |  |  |
| <1 year | 2.26 | 1.88 (0.46-7.69) | 2.74 (0.66-11.29) | - |
| ≥1 year (Ref) | 97.75 | 1.00 | 1.00 | - |
| Knowledge of a partner testing HIV positive in last 30 days |  |  |  |  |
| Knows partners status as negative (Ref) | 69.98 | 1.00 | 1.00 | 1.00 |
| Knows partners status as positive or does not know | 30.02 | 0.69 (0.37-1.28) | 0.82 (0.43-1.59) | 0.22 (0.03-1.73) |

HR = Cox Proportional Hazard Ratio, 95% CI = 95% Confidence Interval

Dashes (-) represent non-convergence due to small sample size

<5% missingness for each variable

**Supplemental Figure 1.** Classification and regression tree analysis of HIV acquisition among complete cases within the full sample (n=423)

**
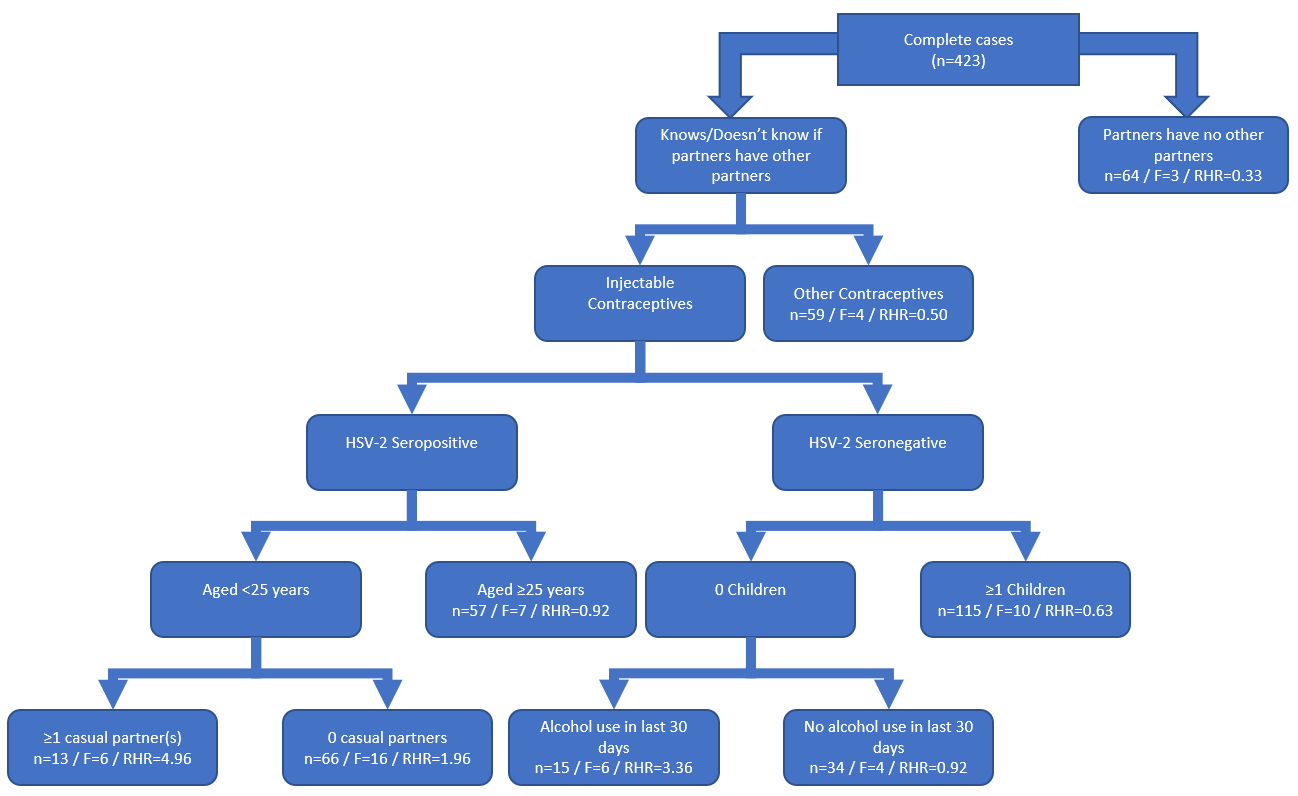
**

Split if adjusted p-value <0.10 using the Chi-square test statistic

Terminal nodes show the number of participants (N), seroconversions (F), and relative hazard ratio (RHR)

Complete classification tree includes complete cases for all variables, resulting in 21 missing cases

**Supplemental Figure 2.** Classification and regression tree analysis of HIV acquisition among complete cases of women <25 years (n=286)


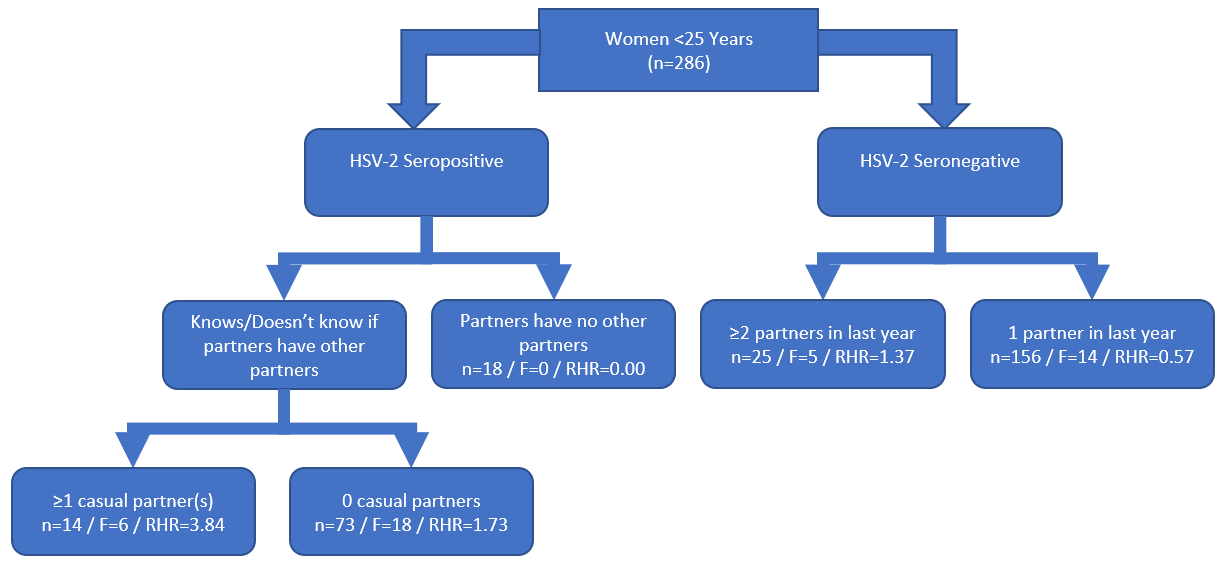


Split if adjusted p-value<0.10 using the Chi-square test statistic

Terminal nodes show the number of participants (N), seroconversions (F), and relative hazard ratio (RHR)

Complete classification tree includes complete cases for all variables, resulting in 16 missing cases

**Supplemental Figure 3: Exploratory multivariable AUC values using highly influential variables: HSV-2, partners have other partners and casual partnerships**

|  | **IRS with only HSV-2 and partners have other partners** | **AIRS with only HSV-2 and partners have other partners** | **MRS with casual partnerships added** |
| --- | --- | --- | --- |
| **ROC curve** | 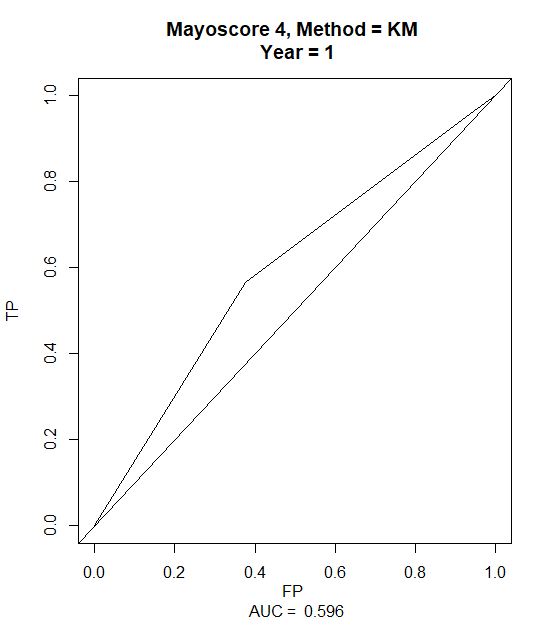 | 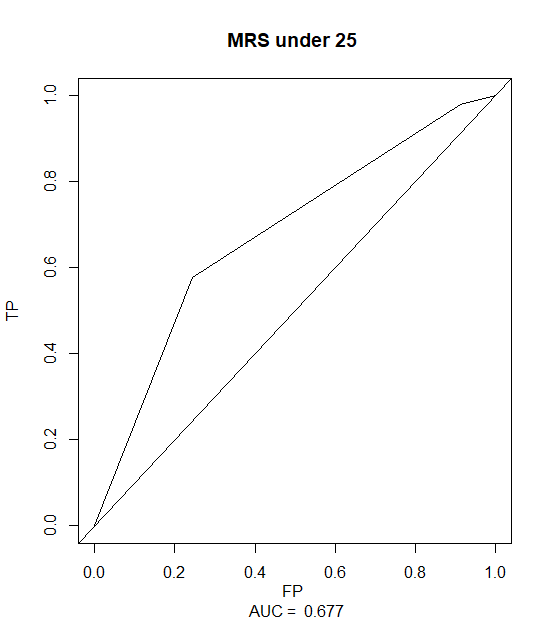 | 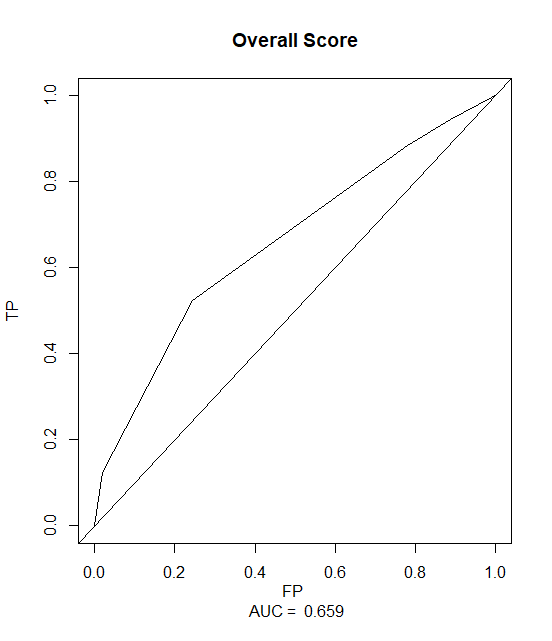 |

ROC = Receiver Operating Characteristic, TP = True Positive, FP = False Positive, AUC = Area Under Curve, IRS = Initial Risk Score, AIRS = Age-stratified Initial Risk Score, MRS = Modified Risk Score

The models in this figure were created by altering existing multivariable risk score models (IRS, AIRS, MRS). In the case of IRS and AIRS, we excluded all factors except HSV-2 and partners having other partners. For MRS, we added casual partnerships to the existing model.
